# Social workers’ intervention during natural hazards

**DOI:** 10.4102/jamba.v14i1.1176

**Published:** 2022-06-29

**Authors:** Frans K. Matlakala, Jabulani C. Makhubele, Louis Nyahunda

**Affiliations:** 1 Department of Social Work, Faculty of Humanities, University of Limpopo, Mankweng, South Africa

**Keywords:** natural hazards, social workers, methods, roles, intervention

## Abstract

Social work profession is anchored on theory and practice and has both primary and secondary methods of interventions. The knowledge base of social work is constituted by values, principles, theories, skills and techniques. Globally, social work scholars have developed paradigms, theories, approaches, perspectives, models, techniques, skills and principles that could be applied in a variety of settings to various social ills for the benefit of clients in communities. Thus, this study was aimed at exploring social workers’ intervention during natural hazards. The researchers used an interpretative qualitative research approach and case study design. Moreover, five social workers who provide psychosocial counselling and social relief of distress were purposively sampled to participate in this study. Data were collected through individual semi-structured interviews and analysed thematically. The study found that social workers use three primary methods of social work: casework, group work and community work. Furthermore, the participants stated that they play roles such as educator, counsellor and broker when dealing with victims of natural hazards. Based on the findings, the researchers have noted that not all social workers are active in providing psychosocial counselling to the victims of natural hazards. As such, the researchers recommend more workshops to educate all social workers that social workers have an important role to perform in the midst of natural hazards.

## Introduction

Social work profession is anchored on theory and practice and has both primary and secondary methods of interventions. The knowledge base of social work is constituted by values, principles, theories, skills and techniques. Globally, social work scholars have developed paradigms, theories, approaches, perspectives, models, techniques, skills and principles that could be applied in a variety of settings to various social ills for the benefit of clients in communities (Zastrow 2017). In light of that, social workers have been using and continue to use their knowledge base when working with groups, individuals and communities. Equally, when faced with natural hazards, the acquired knowledge base can be used to assist victims and survivors. In this study natural hazards will refer to floods, droughts and hail winds that are likely and have occurred at the Greater Tzaneen Municipality. To that end, this article is aimed at identifying intervention strategies that can be used during natural hazards.

### Problem statement

South Africa as a whole, like any other part of the world, is not immune to natural hazards let alone its provinces and municipalities like Limpopo province and Greater Tzaneen Local Municipality. The two most severe, common and observable events in terms of natural hazards in these two areas are floods and droughts. Incidences of meteorological events such as tropical cyclones, tornadoes and extreme rainfall are also not rare. In support of the above observation, the National Disaster Management Centre (NDMC) (2017–2018) reports that South Africa is more likely to be faced with a wider range of hazards, which lead to floods, major fires, tornadoes and even earthquakes (South Africa 2017). Moreover, it is reported that the foundation of disaster predicaments is climate change, which is responsible for the uncontrollable weather conditions globally (Downing & Dow 2006; Green 2009; Bates, Kundzewicz, Wu & Palutikof, 2008; Matlakala 2022; Matlakala, Nyahunda & Makhubele 2021; Nyahunda et al. 2021; Nyahunda, Matlakala & Makhubele 2019; Winker 2010). On the same wavelength, Wallemacq and House (2018) reported that climate-induced natural hazards reckoned for about 90% of the 7255 major natural hazards between 1998 and 2017, most of them floods and storms. Compounded by rising global temperatures, climate-induced natural hazards will become more recurrent and severe, endangering human lives and their livelihoods and putting economic assets at risk (IPCC 2018). To this end, the uncontrollable weather conditions have put vulnerable people at high risks of natural hazards such as tropical cyclones and draughts irrespective of their locations on the globe. Their vulnerability is linked to different social ills ranging from poverty to homelessness. In most instances, people in rural areas, because of their abject poverty and high unemployment rate, which result in their inability to afford house insurance, are rendered by natural hazards to be homeless and to depend on the government for relief.

Woods (2014) indicates that:

[*F*]or disaster management, social work is well located at the nexus between governance and communities so as to promote and facilitate resilience which is now embedded in national disaster management policy. (p. 96)

The challenge is that rural communities do not have enough resources to deal with heavy rains or floods, let alone natural hazards. The only services available to them are of social workers. However, Chanza (2014:2) avers that ‘climate change discourses the collective knowledge, ingenuity and action of all stakeholders (including communities affected by climate change)’. This points to the need to include community members, especially in developing countries and lower classes, in disaster management policies as they are the ones who are mainly affected by natural hazards.

## Research methodology

### Research paradigm and approach

The researchers chose an interpretive paradigm because they were interested in gaining in-depth data on the phenomenon – natural hazards’ services in relation to social work service provision. In support of the assertion, Thomas and Hodges (2010:6) found that qualitative researchers prefer using the interpretative paradigm because it ‘portrays a world in which reality is socially constructed, complex, and ever changing’. On the same note, Neuman (2006) and Thanh and Thanh (2015) pointed out that researchers using the interpretative paradigm use observation and interview for data collection. To that end, the researchers used qualitative data collection methods, that is, individual semi-structured interviews in order to view and make sense of the world using meaning that participants give to the ramification of natural hazards through participants’ own lenses. On the same wavelength, Willis (2007:90) postulated that ‘interpretivists tend to favour qualitative methods such as case studies and ethnography’. Given the nature of this study, the researchers chose an interpretative paradigm as they wanted to adopt a case study as the research design to guide this study.

### Research design

Case study design is an appropriate design that is used to gain contextual and in-depth information about intervention strategies used by social workers in the midst of droughts, floods and heavy storms. According to Simons (2009), case study design examines the experiences of individuals with an aim to understand how individuals put, interpret and give meaning to their situations. Moreover, Beverland and Lindgreen (2010) define a case study as:

[*A*]n exploration of a ‘bounded system’ [*bounded by time and place*] or a case (or multiple cases) over time through detailed, in-depth data collection involving multiple sources of information rich in context. (p. 57)

As indicated above, different authors understand case study design in their own way. However, from the above, it could be deduced that the above authors all agree that case study design is used to explore in-depth phenomenon in its context, using the perspective lens.

#### Exploratory multiple case study design

Considering the qualitative nature of the study, an exploratory multiple case study design was selected. Yin (2014) explains exploratory case study design as a design that attempts to answer the question of ‘what’ in a research. According to De Massis and Kotlar (2014:16), exploratory case study design ‘should be used when the aim is to understand how a phenomenon takes place’. In relation to the study, the researcher was interested in knowing what factors contribute to the emergence of natural hazards in rural areas. Moreover, the researcher was also interested in understanding the roles of social workers and the challenges they face when assisting clients who experienced trauma.

### Targeted population

The total number of the area population cannot form part of the study. It was for that reason that certain population, based on certain demographic factors, was targeted to contribute to the study aim. In this study, the researchers targeted social workers working in rural areas at Tzaneen Municipality. The area was chosen because of its vulnerability to natural hazards as postulated by Greater Tzaneen Municipality (2021), Matlakala et al. (2021) and Matlakala (2022). Moreover, the South African National Disaster Framework –SANDF (2005) calls for the inclusion of various stakeholders in the matter of natural hazards, hence the inclusion of social workers in this study.

### Sampling technique and size

In this study, the researchers used the purposive sampling technique. According to Kumar (2018), purposive sampling is a technique wherein the researcher chooses participants because of qualities that they possess and because they will be able to meet the researcher’s aim. In this study, the researchers purposively selected social workers who dealt with victims and survivors of natural hazards and/or provide Social Relief of Distress (SRD). Greeff (2015) posits that there should be a fair selection of participants by means of inclusion and exclusion criteria depending on the study objective. As such, the pre-selection criterion was that participants had to be working in the Department of Social Development as social workers who deal with SRD within Greater Tzaneen Municipality.

### Sampling size

In spite of the fact that the study was qualitative in nature and its essence was about the depth and richness of the information, a need for sampling size so as to give some guidance through saturation of data was a determining factor. Marshall et al. (2013) assert that in a qualitative study, there is a lack of recognition of sampling size as it is based entirely on the concepts of saturation. For this study, a sample size of five social workers was selected using the purposive technique until the level of saturation.

### Data collection methods

For any empirical research to be completed, data need to be collected. There are various methods to collect data depending on the approach and design of the study. In support of this assertion, Gill et al. (2008) aver that there are a variety of methods of data collection, which include observations, textual or visual analysis (from books or videos) and interviews (individual or group). Moreover, Grix (2010) asserts that data collection is the process through which empirical data are collected via a number of different data sources. The researchers received ethical clearance from Turfloop Research Ethics Committee (TREC). In this study, the researchers used individual semi-structured interviews to collect data from social workers.

### Data collection procedures

#### Individual semi-structured interviews

The researcher conducted individual interviews with social workers for a period of 4 days. Also, to follow health precaution measures put in place by the National Coronavirus Command Council the researchers ensured that all participants wore facemasks, sanitised and maintained 2-m distance during the interviews. Moreover, those with underlying health conditions were requested to declare their condition prior to the interview and were allowed to withdraw from the study without penalty.

### Data analysis method

Data collected from different participants are unorganised and meaningless. Qualitative data analysis is a search for general statements about relationships amongst categories of data, building on grounded theory (Marshall & Rossman, as cited in De Vos et al. 2011). In this study, the researchers used thematic content analysis (TCA) to analyse the data. According to O’Leary (2014), TCA is a method of identifying, analysing and reporting patterns (themes) within the data. It minimally organises and describes data set in (rich) detail. The researchers chose TCA because of its ability and potential to further interpret and highlight emerging themes and aspects pertaining to social workers’ intervention during natural hazards. Moreover, the earlier study by Braun and Clarke (2006) revealed that when using thematic analysis, the researcher has to also indicate if he or she chooses deductive or inductive thematic analysis. In relation to this study, the researcher analysed data using inductive TCA. This method allowed the researcher to develop themes from the emerging research findings.

### Ethical considerations

Ethical clearance to conduct the study was obtained from Turfloop Research Ethics Committee (TREC) (reference number: TREC/358/2019: PG) the Limpopo Provincial Research Ethics Committee (clearance no. LPREC/02/2020: PG). Permission to conduct the study was provided by Limpopo Department of Social Development.

## Findings and discussion

### Social work intervention methods

Social work is a field of study that is not in narrow scope and employs primary methods, namely casework, group work and community for intervention and/or in rendering social services. In support of the above, the National Association of Social Workers (NASW) Delegate Assembly (2008) postulated that because of their broader scope, social workers can utilise their ability and their practice to engage in different methods of social work to advocate for change and transformation. In their practice, social workers are guided by social work methods to enhance social functioning. In their quest to enhance social functioning, social workers work with individuals, groups and communities.

### Casework

Casework is a widely used method of intervention. It is also commonly known as direct practice with individuals. Most social workers use this method when dealing with individuals, couples or families to cope with challenges that impair their social functioning. In this study, participants (social workers) during individual interviews indicated that casework is the most used method of intervention when assisting people who need social work services, be it counselling, SRD, adoption and other services. Likewise, when dealing with individuals who experienced trauma as a result of natural hazards, social workers use the casework method to provide counselling to the victims and survivors of natural hazards. One of the social workers during individual interviews stated that:

‘In my line of duty, depending on the case in point, I use casework because cases have to be treated individually so….’ (Participant 1, Female, Social Worker)

Another social worker stated that:

‘Since I deal with clients who need social relief of distress, the method of intervention that I often use is casework method as it allows a direct contact with the client. In case work situation, the client is able to open up, being given attention individually and there is some degree of trust between me and the client as I ensure confidentiality is maintained.’ (Participant 2, Female, Social Worker)

Social workers believe in the value and principle of individualisation. In the midst of natural hazards, social workers provide psychosocial counselling to individuals to assist them to bounce back to reality. The benefit of social casework or casework was captured by Zastrow (2017) who avowed that an individual solves his or her own problem with the assistance of a social worker. During and after the occurrence of natural hazards, the social workers may use the casework method to assist individuals to unpack the problem at hand, brainstorm on various possible solutions and develop coping mechanisms to solve the challenges that they have without creating dependence. It is through this method that social workers see survivors of natural hazards as experts of their own lives and are able to change and improve their situation. For victims or survivors of natural hazards, empowerment programmes are essential as they assist in broadening the knowledge and facilitating the healing process of individuals. To that end, using this method, social workers respect clients’ right to self-determination, privacy and affirm the worth and dignity of survivors of natural hazards.

### Group work

Individuals in a community experience similar challenges, in this case, trauma, caused by the impact of natural hazards. The process of group work or social group work occurs when two or more people with commonalities working together towards the common goal. In light of this, one of the key participants, a social worker, indicated that group work is an ideal method to provide debriefing session to the victims of natural hazards. The following sentiments were expressed:

‘Sometimes having a support group assist[s] the victim of disasters to cope. I prefer this method because I can manage the group and all the participants get equal opportunity to share their views and all work towards one goal, that is, to change their trauma to strengths.’ (Participant 5, Female, Social Worker)

There is an old adage that ‘two is better than one’. In social work practice, when three or more individuals share a common problem, the social worker opens a social group or groups. In this study, it became evident that in the midst of natural hazards, social workers prefer a support group in the form of a debriefing session. Kirst-Ashman (2017) opined that the benefit of social group work is that individuals freely express their feelings and share their coping strategies about natural hazards, whether man-made or natural. In essence, the visibility of the support group is seen as another method that can be used during natural hazards. This is because Africans are gregarious in nature and when natural hazards erupt, the embedded philosophy of *Ubuntu* automatically kicks in; they support each other. As such, social support groups could be established to discuss coping skills with survivors and victims of natural hazards.

### Community work

In rural areas, most of the community members are illiterate and depend solely on the traditional knowledge for their survival. On that note, social workers adopt community work models such as social marketing and education in order to notify community members of the service they are providing and also to educate them about the importance of protecting the environment. In this study, one of the key informants, a social worker, stated that the community work method is mostly used as a disaster preparedness technique. In elaborating, one of the social workers averred that:

‘We do awareness campaign and have community dialogue to inform community members of the services offered by social workers.’ (Participant 3, Male, Social Worker)

Social workers operate within a wider spectrum and those they serve sometimes remain in the dark. As such, social workers use their community work models in order to create awareness and discuss their tasks, roles and functions in communities. Likewise, with natural hazards, the participants indicated that they use awareness campaigns in order to inform community members, including small-scale farmers, about the rampage of natural hazards and its undesirable ramifications. Dhavaleshwar and Banasode (2017) underscored that community work is the core of social work education as social workers use this method to help discover and rebuild community resilience after natural hazards. When community resilience is identified, enhanced or restored, community members are able to effectively prepare, respond and recover from natural hazards. To that end, Manyena (2006) opined that without a doubt, successful adaptation to mitigate the effects of natural hazards depends on collective and individual efforts.

### Social work roles

Social work practice plays a significant role in society as it aims to enhance the social functioning of individuals, groups and communities. In their intervention, social workers are guided by various roles regardless of challenges they face during natural hazards.

#### Educator

Rural areas are populated by individuals with high illiteracy. Given the wide scope of their services, social workers have a role to play in ensuring that they empower and educate community members on the impact of natural hazards. In essence, social workers should provide community members with adaption and response strategies that could be used in the face of natural hazards (Kirst-Ashman 2010). In providing ways to mitigate and respond to climate change, one of the key participants, a social worker, indicated that they use the community education model to educate the community about residing in areas that are prone to natural hazards. The social worker said that:

‘We educate the community members about the danger of residing in areas that are not safe, like staying next to the river. We provide them with this knowledge to make sure that our community members do not predispose themselves to the risks of disasters….’ (Participant 1, Female, Social Worker)

Another social worker stated that:

‘We provide awareness campaign to community members where we educate community members about the significance of remaining calm during disasters.’ (Participant 3, Male, Social Worker)

Rurality serves as one of the contributing factors that predispose community members to natural hazards. In all of that, social workers assume the role of an educator where they educate people about the danger of residing in areas that are prone to natural hazards. Sheafor and Horejsi (2012) stated that the role of an educator or teacher is to facilitate behavioural change and primary prevention. In other words, the social worker can use this role to modify and change a negative behaviour of clients. In terms of community, the social worker can teach community members about the effects of climate change so that they can change their behaviour about their environment and conserve it. In relation to this study, social workers have to teach the community members coping, adaptation and responding skills that would make them able to mitigate the effects of natural hazards. In so doing, they will also be rebuilding community resilience amongst community members and position community members to co-exist and support each other in the midst of natural hazards.

#### Counsellor

Natural hazards disrupt the normal functioning of individuals, families and communities. As a result, the aftermath of natural hazards is characterised by feelings of depression, post-traumatic stress disorder and anxiety. It is during this unpleasant event that community members require professional assistance to develop coping strategies during difficult times. In social work practice, social workers assume the role of a counsellor to provide counselling to the victims and survivors of natural hazards (Kirst-Ashman 2017). In this study, it became evident that social workers provide psychosocial counselling to families and children when they are terrified by the impact of natural hazards on their livelihood. One of the social workers stated that:

‘I provide psychosocial counselling to the victims and survivors of disasters and try to help them to bounce back to the state of normality.’ (Participant 2, Female, Social Worker)

Undoubtedly, community members’ livelihood is affected by the impact of natural hazards. As a result, some of the individuals are left with psychological distress, whilst others are left homeless. In this study, the participants acknowledged that after a disaster community members were provided with psychosocial counselling. In most instances, social workers use casework, because it is clinically orientated, to assist an individual in coping, responding and adapting at the aftermath of natural hazards. In so doing, social workers will be building individual resilience so that individuals can bounce back to normality and use their experience to prepare for other natural hazards should they occur.

#### Broker

Social workers assist all individuals regardless of their socio-economic status. Sometimes clients that come into their office seek services that are above the scope of social work. For instance, during natural hazards, people lose their personal documentations and seek assistance from social workers. In those instances, social workers link those clients with the relevant agencies, such as the Department of Home Affairs. Likewise, in this study, the researcher found that social workers assume the role of the broker during and after experiencing natural hazards. The following sentiments were shared by one of the social workers who stated that:

‘As social workers, those who lost their shelter and placed in temporary accommodation during disaster, we link them with the Department of Human Settlement.’ (Participant 2, Female, Social Worker)

Another participant, a social worker, indicated that:

‘I have referred the victims of disaster to the Department of Home Affairs because their vital documents were damaged also.’ (Participant 4, Female, Social Worker)

Social workers assume the role of brokers and link them with the relevant department that will cater for their needs. Kirst-Ashman (2017) defines the broker as a person who assists in linking clients with community resources and services. In this study, it was revealed that social workers link those who lost their identity documentation because of natural hazards to the Department of Home Affairs to access service. Sheafor and Horejsi (2012) suggested that before linking the client, the social worker should assess the client’s vulnerability, culture, abilities and the intelligence of the client. In this study, lack of documentation means children will be unable to register with institutions, and the elderly will fail to apply for SRD during and after natural hazards. Hence, Sheafor and Horejsi (2012) insist that social workers should be knowledgeable about the agencies around them to avoid referring clients to a wrong agency. Fortunately, in this study, the participants were well equipped and linked the victims of natural hazards to relevant departments.

## Conclusion and recommendations

In this study, it became evident that social workers have a role to play during natural hazards. Social workers stated that they prefer three primary methods of intervention: casework, group work and community work. It was to be expected that all the three methods will be the preferred ones as most community members seek all sort of help individually after natural hazards. To be precise, those affected by natural hazards need psychosocial counselling and social workers indicated that they prefer offering it individually. In contrast, other social workers have recognised the importance of using the group work method and later employ community models. Within those methods, social workers also perform different roles, such as educator, counsellor and broker, to those affected by natural hazards. In light of that, the researchers call for more workshops to educate all social workers that they have an important role to perform in the midst of natural hazards. Social workers should work with other stakeholders during and after natural hazards in the quest to enhance social functioning of the social recipients.
